# From Lyme disease emergence to endemicity: a cross sectional comparative study of risk perceptions in different populations

**DOI:** 10.1186/1471-2458-14-1298

**Published:** 2014-12-18

**Authors:** Cécile Aenishaenslin, André Ravel, Pascal Michel, Lise Gern, François Milord, Jean-Philippe Waaub, Denise Bélanger

**Affiliations:** Groupe de Recherche en Épidémiologie des Zoonoses et Santé Publique (GREZOSP), Pavillon de la santé publique, Faculté de médecine vétérinaire, Université de Montréal, CP 5000, Saint-Hyacinthe, J2S 7C6 Québec Canada; Laboratory for Foodborne Zoonoses, Public Health Agency of Canada, CP 5000, Saint-Hyacinthe, H2S 7C6 Québec Canada; Laboratoire d’Eco-Epidémiologie, Institut de Biologie, Université de Neuchâtel, 11 Emile-Argand, CP 158, 2009 Neuchâtel, Suisse; Institut national de santé publique du Québec, 1255 Beauregard, Longueuil, J4K 2M3 Québec Canada; Groupe d’étude et de recherche en analyse de la décision (GERAD), 3000 Côte-Sainte-Catherine, Montréal, H3T 2A7 Québec Canada

**Keywords:** Lyme disease, Lyme borreliosis, Risk perception, Prevention, Preventive behaviors, Knowledge, Emergence, Endemicity, General public, Experts

## Abstract

**Background:**

Lyme disease (LD) is a tick-borne emerging disease in Canada that has been endemic in many temperate countries for decades. Currently, one of the main approaches for LD prevention is the promotion of individual-level preventive behaviors against ticks. Health behaviors are influenced by individual and social factors, one important of which is risk perception. This study aims to describe and compare risk perception of LD, within and between general populations and experts living in two different regions: the Neuchâtel canton in Switzerland, where LD is endemic, and the Montérégie region in Québec (Canada), where LD is emerging.

**Method:**

A web-based survey was conducted in both study regions (814 respondents) in 2012, and a questionnaire was administered to 16 experts. Comparative analyses of knowledge, risk exposure and different components of LD risk perception were performed. Multivariate analyses were used to calculate a global risk perception score and to identify determinants of risk perception in both regions.

**Results:**

In Montérégie, only 15% of the survey respondents had a good level of knowledge of LD compared to Neuchâtel where 51% of survey respondents had good levels of knowledge. In Montérégie, 24% of respondents perceived themselves as being at *high* or *very high risk* of contracting LD *vs* 54% in Neuchâtel; however, a higher percentage of respondents from this region believed that personal protection was simple to carry out (73% *vs* 58% in Montérégie). Based on the population surveys, almost all of the identified determinants of risk perception were different between both populations except for gender. A good level of knowledge, living in the risk zone and knowing someone who has had LD increased risk perception, while a high level of education and being 18–34 years of age decreased this perception. The majority of the studied components of risk perception were different between populations and their regional experts.

**Conclusion:**

This study suggests that risk perception of LD differs between populations and regional experts living in different epidemiological situations. Monitoring of knowledge and risk perception in local populations may help to better target LD communication efforts in accordance with population specific attributes thereby enhancing prevention efficacy.

## Background

Lyme disease (LD), or Lyme borreliosis, is the most frequent vector-borne disease in temperate countries [1]. In most cases, the disease causes non-specific flu-like symptoms and a typical skin lesion known as *erythema migrans*. More severe systemic infections can occur in some cases, and may lead to arthritis, cardiac and neurological problems [2]. With a recent adjusted estimation of about 300,000 human cases annually in the United States [3] and about 85,000 cases in Europe [1], Lyme disease is a growing concern in many countries, including Canada where it is currently emerging. In the province of Québec (Canada), locally acquired cases were first identified in 2008 [4]. Populations of black-legged ticks (*Ixodes scapularis*), the only known vector of Lyme disease on the eastern-American coast, are now recognized as established in the southern part of the province, in the Montérégie region. In this region, 8-13% of the black-legged ticks have been found to be infected with *Borrelia burgdorferi*, the bacteria causing LD [5]. In Switzerland, LD cases have been reported for more than 30 years [6]. The disease has not been notifiable since 2003, but current estimates place this country third highest for LD incidence in Europe with 83 cases per 100,000 inhabitants reported in 2010 [7]. In this country, *Ixodes ricinus* is the vector responsible for the transmission of LD, and prevalence of *Borrelia burgdorferi* infection in ticks is as high as 40% in some regions [8]. Moreover, in several regions of the country, these ticks are known to carry tick-borne encephalitis virus (TBEV), the agent of tick-borne encephalitis (TBE), another severe and notifiable disease in Switzerland [9].

Although LD ecology differs in Europe and North America with regards to the importance of different reservoir species of the bacteria and the primary tick vector involved in transmission [10], the main preventive strategy is the same in both regions and relies primarily on individual-level preventive behaviors [11]. Preventive behaviors such as checking for ticks after visiting affected wooded regions, wearing long trousers or repellent containing DEET, have been shown to be efficient in the prevention of LD [12–18]. However, beyond their demonstrated efficacy, studies have also shown that people do not apply these measures with the same consistency, even in highly prevalent regions [19–29].

Predictors of individual-level preventive behaviors have been studied for many health conditions. The Health Belief Model is one widely used theoretical model developed to study health behaviors. In this model, one main determinant of a health behavior is the perception of risk, defined as the subjective assessment of the probability and the consequences of a specified type of hazard [30]. Risk perception is composed of the perceived severity of and the perceived susceptibility to the disease in question [31]. This model has been validated for many diseases and health conditions including LD, for which a higher level of risk perception was associated with an increased adoption of preventive behaviors [19, 28, 29, 32–34]. As a result, risk perception has become a major point of interest for decision-makers involved in the design and the implementation of preventive communication programs. An extensive literature exists on risk perception, Paul Slovic being a pioneer of the psychometric approach, which recognizes that risk perception is a construct reflecting individual and social level influences [35–37]. Studies have shown tendencies which seem to persist among different fields of research: the perceived risk in the general public differs from the risk as perceived (or evaluated) by experts [38]; determinants of risk perception are numerous and multidimensional, and they include characteristics of the hazard in question such as the novelty of the hazard and its potential catastrophic impacts, as well as individual and sociological factors, such as gender, age, education, income, personality, culture and values [35, 39–41].

Past studies have described LD risk perception in particular regions or countries [19, 21, 22, 25, 27–29, 32], but none have explored the differences between the determinants of risk perception in different epidemiological contexts, such as in a population experiencing the emergence of LD versus a population that has been living in a region endemic for LD during a long period of time. Are determinants of LD risk perception universal, or do they vary according to the context, such as the epidemiologic situation? The identification of context-specific determinants of LD risk perception would provide additional insights for decision-makers in the planning of LD risk communication that could be better adapted to emerging or endemic situations. Moreover, it could help decision-makers in emerging contexts to anticipate the changes in their population’s risk perception that may occur once LD becomes endemic.

With this perspective in mind, the main objective of this study was to compare risk perception of LD and to describe its determinants within and between two different populations: residents of the Neuchâtel canton, in Switzerland where LD has been endemic for more than 30 years, and residents of the Montérégie region, in Québec, Canada, where LD is emerging and where the indigenous cases were first reported in 2008. A second objective was to compare perceptions of the general population with perceptions of regional LD experts, and between experts from both regions. Estimated LD incidence in the Neuchâtel canton ranged from 49 to 95 cases per 100,000 inhabitants by 1996–2001 [42, 43], which was above the national mean incidence for Switzerland. Montérégie had an estimated incidence of 0.5 cases per 100,000 inhabitants in 2012, making it the most affected region in the province of Québec (Canada) [44].

## Methods

### Study design

This cross sectional study was based on a web survey that was administered simultaneously in the fall of 2012 in the two study regions. The questionnaire, which included 58 questions, was constructed for the purposes of this study and was based on the theories of health behaviors [31] and on existing questionnaires measuring LD knowledge, attitudes and behaviors [45, 46]. Questions were designed to measure perceptions of LD: perceived severity, perceived individual susceptibility, perceived susceptibility for other residents in the region (perceived regional susceptibility), perceived personal control on LD prevention (perceived mastery), perceived scientific uncertainty (perceived uncertainty), perceived confidence in preventive public programs, feeling of worry, perceptions of the efficacy of preventive individual-level and environmental-level measures, and social acceptability of preventive environmental measures. Moreover, LD knowledge (four items were evaluated: knowledge related to the transmission mode, early symptoms, treatment, risk zone), frequency of exposure through outdoor activities (used as an indicator of the level of exposure), past experiences with LD (knowing someone with LD or having had LD before), adoption of individual preventive behaviors and socio-demographic characteristics (gender, age, education, family income, geographic location of residency) were also included. Questions pertaining to perception were evaluated using a five point Likert scale: (5) strongly agree, (4) agree, (3) neither agree nor disagree, (2) disagree, (1) strongly disagree. In order to allow participants with no knowledge of LD to complete the questionnaire, all participants were given a short informative text providing general basic information about LD as provided by government websites in both regions (excluding the knowledge questions which were administered before this reading and only to those having declared that they had previously heard of LD). The questionnaire and the descriptive text accompanying it were adapted for the Canadian and Swiss contexts. The general content of the questionnaires was the same for both regions; however, the exact wording of some questions was adapted to account for cultural differences, such as family income and education levels, and for three specific items which were added to the Neuchâtel questionnaire: the perceived knowledge of Tick-Borne encephalitis (TBE), the perceived knowledge of differences between LD and TBE, and the district of residency. There are six districts in the Neuchâtel region: three of the districts (Neuchâtel, Boudry, Val-de-Ruz) are at a higher risk of LD (low altitude) compared to the other three districts (Val-de-travers, Le Locle, La-Chaux-de-Fond) considered to be at lower risk due to their location at higher altitudes where tick densities are lower [43, 47]. The questionnaire was designed to study perceptions, attitudes and preventive behaviors in a global perspective. This paper will focus on knowledge, risk perception and its determinants.

The questionnaire was developed and administered in French, which is the main language of both the Montérégie and Neuchâtel regions. It was pre-tested to verify the formulation of questions and general understanding with 35 people from the general public through focus groups conducted between August 12^th^ and September 30^th^ 2012 in both regions. This protocol was reviewed and approved by the Ethical Committee for Health Research of the University of Montreal (CERES).

### Data collection

In each region, the questionnaire was administered to a random sample of members of a web panel. The panels used in this study are administered by the external survey firm Leger Marketing [48]. They include individuals from the general population who had engaged either voluntarily with the panel or had been recruited through probabilistic phone surveys by the firm in order to be representative of each region in terms of socio-demographic factors including gender, age, education, income and geographic distribution. These panels are used to complete surveys on a large variety of subjects which are not related to Lyme disease including research, marketing studies and opinion polls and are a good representation of the general public for our study object. In order to reach a sample size of 400 participants in each region, the invitation to participate was sent to two subsamples of the regional web panels, for a total of 5,222 people in Montérégie and of 1,233 people in Neuchâtel, which represent respectively 0.4% and 0.7% of the total population [49, 50]. Inclusion criteria were: to be 18 years of age or older, to be a resident of one of the two study regions and to understand French. The survey was available online from November 19^th^ to December 1^st^ 2012 in Neuchâtel and from November 19^th^ to November 22^nd^ 2012 in Montérégie (it was closed when 400 respondents had completed it).

Another questionnaire was designed for experts using a subset of questions from the main questionnaire in order to measure risk perception, perceptions of the efficacy of individual and environmental level preventive measures, and perceived acceptability of environmental measures. Experts were selected based on their involvement in LD management. In Montérégie, seven experts were invited to complete the questionnaire and were those who had previously participated in a study for LD management in Québec [51]. In Neuchâtel, nine experts were invited and represented members of the National Reference Centre for tick-transmitted diseases [52]. The expert questionnaires were sent by e-mail and the response period for both regions was between August 12^th^ and September 30^th^ 2012.

### Data analysis

Statistical analyses were stratified for each study region and performed using IBM SPSS Statistics 19. Confidence intervals for proportions were computed using the Clopper-Pearson method with a confidence level of 95% [53]. Pearson Chi-square statistics were performed to detect significant differences (p < 0.05) between groups (study regions, gender, age groups). A global knowledge score (null, medium, high) was developed based on the four items assessed (for a maximum of four possible good answers): (null (0 = no good answer), medium (1 = 1 or 2 good answers) and high (2 = 3 or 4 good answers)). Participants who declared that they have never heard about LD before the survey were automatically given 0 for this score.

To allow for descriptive comparisons, mean scores, modes and ranges were calculated for a selection of perception variables for both the population and for the expert surveys. Statistical differences between mean population scores were tested using the Student’s t test for independent samples with p < 0.05.

In order to select the most important perception variables and be able to summarize them in a global risk perception score for each participant, an exploratory factor analysis (EFA) [54] was performed and initially included seven risk perception variables (perceived severity, perceived individual susceptibility, perceived regional susceptibility, perceived mastery, perceived uncertainty, perceived confidence, feeling of worry). Factor extraction was performed using the unweighted least squares method (recommended for ordinal data) and an oblique rotation (recommended for psychosocial measures) [55, 56]. EFA necessitates that a sufficient correlation exist between variables that are included in the analysis. Therefore, variables with an initial quality of representation inferior to 0.2 (i.e. the part of the variable variance that can be explained by all other variables) were excluded from the analysis. Factors with eigenvalues under 1 were not considered. Selected variables were those with factor loadings on the perception factor that were superior to 0.5. A Kaiser-Meyer-Olkin measure of sampling adequacy was calculated for both regions [57]. For each participant, a global risk perception score was then calculated as the mean score of the selected variables based on the results of the factor analysis. Cronbach alpha was calculated using the selected perception variables on the total sample, as an indicator of the internal consistency of the perception measures [58].

Linear multivariate regressions were then performed separately for the two regional subsets using the global risk perception score as the dependant variable. Multivariate analyses were stratified by region because we anticipated that predictors of risk perception would be different in both contexts. Univariate regressions were done separately for each independent variable (age, gender, household income, education level, general knowledge about LD, region of residency, individual frequency of exposure in public area, personal history with LD, history with LD in relatives) and variables associated with the dependant variable with p < 0.20 were included in the initial multivariate models. Reduced final models were selected using a backward elimination process with p < 0.05. After the identification of significant predictors in each separate region, these were forced in the other region’s model as independent variables, even if not significant, in order to allow proper comparisons of the coefficients between populations.

## Results

### Sample description

A total of 814 participants completed the questionnaire (401 in Montérégie and 413 in Neuchâtel), for a combined response rate of 14%. In Montérégie, 199 (50%) participants were women, 191 (48%) were 55 years old or more, 168 (42%) had a level of education equivalent to college and 135 (34%) had a family income between 40,000 and 79,999 $CAN. In Neuchâtel, 241 (58%) participants were women, 112 (27%) were 55 years old or more, 209 (51%) had a college level of education and 139 (34%) had a family income between 40,000 and 79,999 CHF (Table 1). The distribution of these socio-demographic characteristics were similar to the underlying populations when compared to regional census data [49, 50].

**Table 1:** **Sociodemographic description of the 814 participants by study region**

	Montérégie	Neuchâtel
	n (%)	n (%)
Total	401 (100)	413 (100)
Gender		
Women	199 (50)	241 (58)
Men	202 (50)	172 (42)
Age		
18-34 yr	57 (14)	110 (27)
35-54 yr	153 (38)	191 (46)
55+ yr	191 (48)	112 (27)
Education level		
High school or less	113 (28)	28 (7)
College or equivalent	168 (42)	209 (51)
University or equivalent	112 (28)	170 (41)
na*	8 (2)	6 (1)
Household income ($CAN or CHF)		
<40 000	83 (21)	54 (13)
40 000–79 999	135 (34)	139 (34)
80 000–119 999	88 (22)	103 (25)
> or = 120 000	29 (7)	43 (10)
na*	66 (17)	74 (18)

*Prefer not to answer.

### Past history with LD, exposure and knowledge

In Montérégie, 185 (46%) participants declared that they have never heard of LD before the survey, 14 (4%) knew someone who had contracted LD and 3 (1%) declared that they had previously had LD compared to 89 (22%), 168 (41%) and 24 (6%) of participants respectively in the Neuchâtel region (proportions are all significantly different between regions with p < 0.0001; Table 2).

**Table 2:** **Descriptive analysis of past history with LD, exposure, knowledge and perceptions per region**

	Montérégie		Neuchâtel	
	n	% (CI) ^1^	n	% (CI) ^1^
Total	401		413	
Past history with LD				
Know someone with LD	14	3 (2–6)	168	41 (36–46)*
Have ever had LD	3	1 (0–2)	24	6 (4–9)*
Have a dog	83	21 (17–25)	74	18 (14–22)
Never heard about LD	185	46 (41–51)	89	22 (18–26)*
Know LD for one year or less	42	10 (8–14)	48	12 (9–15)
Know LD for more than one year	174	43 (38–48)	276	67 (62–71)*
Exposure frequency through outdoor activities				
Less than 2 times per yr	177	44 (39–49)	47	11 (8–15)*
2-10 times per yr	155	39 (34–44)	155	38 (33–42)
11-25 times per yr	41	10 (7–14)	97	23 (19–28)*
More than 25 times per yr	28	7 (5–10)	114	28 (23–32)*
Knowledge on LD				
Transmission mode (Know that LD is transmitted by a tick bite)	112	28 (24–33)	270	65 (61–70)*
Early symptom (Know that skin erythema is an early sign of LD)	115	29 (24–33)	224	54 (49–59)*
Treatment (Know that LD can be treated with systemic antibiotics)	71	18 (14–22)	182	44 (39–49)*
Risk zone (Know where it is possible to contract LD in their region)	72	18 (14–22)	228	55 (50–60)*
Global level of knowledge				
High (% with global score of 3 or 4)	60	15 (12–19)	209	51 (46–56)*
Medium (% with global score of 1 or 2)	117	29 (25–34)	105	25 (21–30)
Null (% with global score of 0)	224	56 (51–61)	99	24 (20–28)*
Specific items related to TBE (Neuchâtel only)				
Never heard about TBE	-	-	75	18 (15–22)
Know TBE for one year or less	-	-	36	9 (6–12)
Know TBE for more than one year	-	-	302	73 (69–77)
Know well the differences between TBE and LD (self-declared % of scores 4–5 on an agreement scale)	-	-	95	23 (19–27)
Risk perceptions (% with score 4–5)				
High-perceived individual susceptibility	95	24 (20–28)	223	54 (49–59)*
High-perceived regional susceptibility	163	41 (36–46)	234	57 (52–61)*
High-perceived severity of LD	304	76 (71–80)	328	79 (75–83)
High-feeling of worry	99	25 (21–29)	149	36 (31–41)*
High-perceived mastery	231	58 (53–62)	301	73 (68–77)*
High-perceived uncertainty	177	44 (39–49)	89	22 (18–26)*
High-perceived confidence	89	22 (18–27)	176	43 (38–48)*
Global risk perception score (% with score ≥ 4)	77	19 (15–23)	141	34 (30–39)*

^1^95% confidence intervals (Exact binomial Clopper-Pearson Method).

*p < 0.0001 (Pearson Chi-square).

In Neuchâtel, 51% (211/413) of participants declared having had a high-level of exposure (10 or more outdoor activities in a LD risk region during the risk period), compared to 17% (69/401) in Montérégie, which is significantly lower (p < 0.0001) (Table 2). In Neuchâtel, highly exposed respondents were primarily women (120/211, 57%), aged 35–54 years old (93/211, 44%), with a high level of knowledge of LD (124/211, 59%), whereas in Montérégie, men were the most highly exposed group (44/69, 64%), aged 35–54 (33/69, 48%), with only 17% (12/69) reporting a high level of LD knowledge.

**Figure 1:**
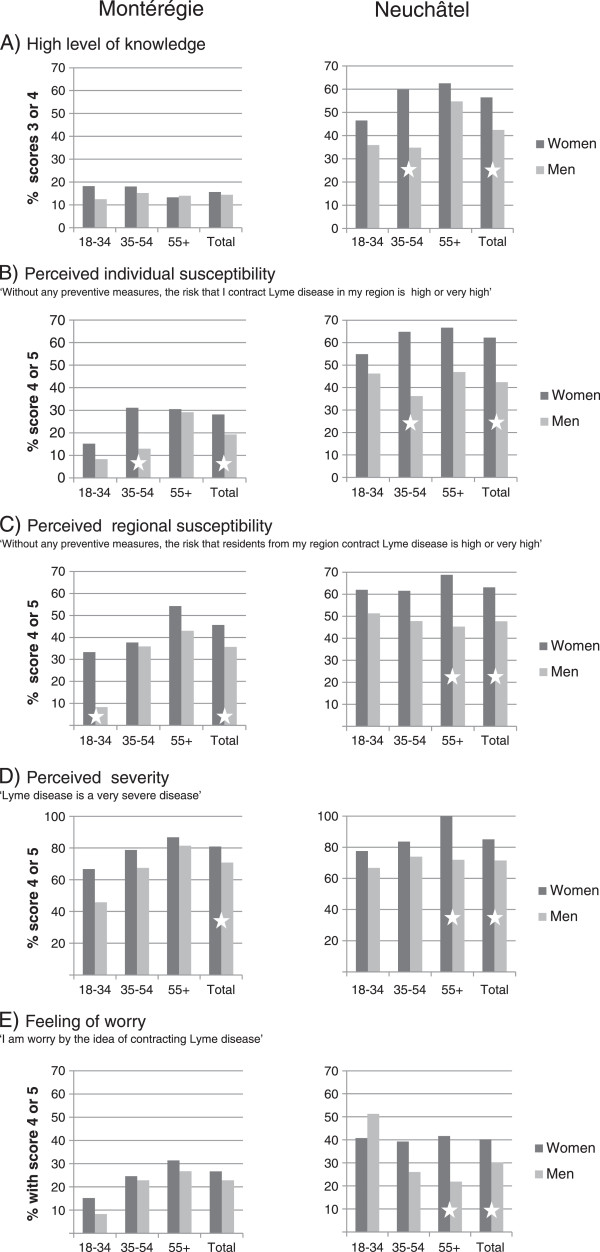
**Distribution of high levels of knowledge (A), perceived individual susceptibility (B), perceived regional susceptibility (C), perceived severity (D) and feeling of worry (E) in both regions, according to gender and age groups (dark gray represents proportions in women and light gray in men; stars represent significant differences in proportions between women and men in different age groups and globally).**

The proportion of respondents with a high level of knowledge of LD was significantly higher in Neuchâtel with 51% (209/413) compared to 15% (60/401) in Montérégie (p < 0.0001). Proportions of good answers on the four knowledge questions ranged from 44 to 65% in Neuchâtel, and from 18 to 29% in Montérégie (Table 2), the most commonly failed questions being on LD treatment and knowledge of risk zones. Figure 1A presents the proportions of participants with high levels of knowledge by gender and age group. In Neuchâtel, globally, high-levels of knowledge was more frequent in women (136/241 or 56% *vs* 73/172 or 42% in men, p = 0.005) with the greatest disparities in the group of 35–54 year olds (73/122 or 60% for women *vs* 24/69 or 35% for men). In men, the proportion was higher in the 55+ yr olds *vs* other age groups (p = 0.01). There was no significant difference between age groups in women. In Montérégie, the proportion of respondents with a high-level of knowledge remained low within gender and age groups with no significant differences (p ≥ 0.05) (Figure 1).

### Risk perception within and between regions

Globally, the proportion of respondents with a high level of risk perception (% of respondents with scores of 4 or 5) was greater in Neuchâtel for all perception variables, except for the *perceived severity* (Table 2). The proportion of respondents with high-perceived mastery was also higher in Neuchâtel (73% or 301/413 *vs* 58% or 231/401 in Montérégie, p < 0.001). Within both populations, the proportions of respondents with high scores for the *perceived individual susceptibility*, the *perceived regional susceptibility*, the *perceived severity* and for *feeling of worry* varied according to gender and age groups (Figure 1, B, C, D and E). In both regions, proportions were significantly greater in women for all of these items (p < 0.05), except for *feeling of worry* in Montérégie. In Montérégie, for all four variables, the proportion of respondents with high-perceived risks was different between age groups (p < 0.05), while no significant difference was identified between age groups in Neuchâtel. In Neuchâtel, 23% (95/413) of respondents declared that they had a good knowledge of the difference between TBE and LD (score of 4 or 5 on the agreement scale) and 18% (75/413) had never heard about TBE.

Considering central tendencies, the mode was consistent between regions for four perception variables: the *perceived regional susceptibility* (mode = 4), the *perceived severity* (mode = 4), the *perceived mastery* (mode = 4) and the *perceived confidence* (mode = 3), but differed for the *perceived individual susceptibility* (mode = 2 in Montérégie *vs* 4 in Neuchâtel), the *perceived uncertainty* (mode = 4 in Montérégie *vs* 3 in Neuchâtel) and *feeling of worry* (mode = 2 in Montérégie *vs* 3 in Neuchâtel) (Table 3). *Perceived individual susceptibility* means were 2.7 in Montérégie and 3.4 in Neuchâtel, and were both smaller than the mean *perceived regional susceptibility*, which was equal to 3.1 and 3.6, respectively. All mean scores were significantly different between populations (p < 0.05, Table 3).

**Table 3:** **Comparison of mean scores and modes for seven perception’s dimensions between the general population and their regional experts**

	Montérégie		Neuchâtel	
	Population	Experts	Population	Experts
Total	401	7	413	9
**Perceived individual susceptibility**				
Without any preventive measures. the risk that I contract Lyme disease in my region is high^1^				
Mean score	2.7	-	3.4***	-
Mode	2	-	4	-
**Perceived regional susceptibility**				
Without any preventive measures, the risk to contract Lyme disease for residents is high				
Mean score	3.2	1.9	3.6***	3.9
Mode (range)	4	2 (1–3)	4	4 (2–5)
**Perceived severity**				
Lyme disease is a very severe disease				
Mean score	4.0	3.0	4.1*	3.7
Mode (range)	4	3 (2–4)	4	3 (3–5)
**Perceived mastery**				
It is easy to protect myself against Lyme disease				
Mean score	3.6	4.9	3.9***	4.7
Mode (range)	4	5 (4–5)	4	5 (4–5)
**Perceived uncertainty**				
I have the feeling that there is great scientific uncertainty concerning Lyme disease				
Mean score	3.3	2.7	2.7***	3.8
Mode (range)	4	2 (2–4)	3	4 (3–5)
**Feeling of worry**				
I am worry by the idea of contracting Lyme disease				
Mean score	2.7	-	3.0***	-
Mode	2	-	3	-
**Feeling of confidence**				
I am confident that responsible authorities set up appropriate measures to control Lyme disease				
Mean score	3.1	3	3.4**	3.2
Mode (range)	3	2 (2–5)	3	4 (2–4)
**Global risk perception score** ^**2**^				
Mean score	3.2	-	3.5***	-
Mode	2.8	-	3.8	-

^1^All measurement scales are 1: strongly disagree, 2: disagree, 3: neither agree or disagree, 4: agree, 5: strongly agree.

^2^Global risk perception scores represent the mean score on: perceived individual susceptibility, perceived regional susceptibility, perceived severity of the disease and feeling of worry.

*p < 0.05;**p < 0.01; ***p < 0.001 (Student t test).

### Risk perceptions in experts

Except for the *perceived regional susceptibility* in Neuchâtel, the modes differed between populations and experts for all other measured perception variables (Table 3). With regards to inter-regional expert comparison, modes differed on three variables: the *perceived regional susceptibility* (experts mode = 2 in Montérégie *vs* 4 in Neuchâtel), the *perceived uncertainty* (experts mode = 2 in Montérégie *vs* 4 in Neuchâtel) and *the perceived confidence* (experts mode = 2 in Montérégie *vs* 4 in Neuchâtel).

### Factor analysis and global risk perception score

For both regional subsets, first EFA led to the exclusion of two variables with a quality of representation inferior to 0.2: *perceived uncertainty* and *feeling of confidence* (Table 4). Final EFA suggested the presence of one latent factor with an eigenvalue superior to 1, with four main contributing variables: *perceived individual susceptibility*, *perceived regional susceptibility*, *perceived severity* and *feeling of worry* (*perceived mastery* was excluded because its factor loading on the factor was inferior to 0.5). These variables were the same for both regions, with factor loadings ranging from 0.51 to 0.80 in Montérégie and from 0.52 to 0.84 in Neuchâtel (Table 4). The Kaiser-Meyer-Olkin measure of sampling adequacy was 0.714 in Neuchâtel and 0.762 in Montérégie, which is considered as acceptable [57]. The final models explained 42.0% and 48.5% of variance in Neuchâtel and Montérégie, respectively. Cronbach alpha for the four variables was 0.760 which is considered acceptable (calculated on the total sample, n = 814). The latent factor was interpreted as the global risk perception, and the four contributing variables were selected to construct the global risk perception score (mean score of the four selected variables). The global risk perception score ranged from 1 to 5, with a mean of 3.2 in Montérégie and of 3.5 in Neuchâtel (Table 3).

**Table 4:** **Exploratory factor analysis of the perception variables**

	Montérégie	Neuchâtel
**Initial model**		
Quality of representation^1^ of EFA with seven variables (used for initial selection of variables)		
Perceived individual susceptibility	0.47	0.40
Perceived regional susceptibility	0.66	0.67
Perceived severity	0.27	0.30
Feeling of worry	0.62	0.44
Perceived mastery	0.22	0.34
Perceived confidence^2^	0.12	0.08
Perceived uncertainty^2^	0.04	0.07
**Final model**		
Percentage of variance explained	48.5	42.0
Kaiser-Meyer-Olkin measure of sampling adequacy	0.762	0.714
Factor loadings of retained variables^3^		
Perceived individual susceptibility	0.67	0.63
Perceived regional susceptibility	0.80	0.84
Perceived severity	0.51	0.52
Feeling of worry	0.76	0.54
Cronbach alpha (total sample)	0.760	

^1^The quality of representation represents the variable variance that can be explained by all other variables.

^2^Variables excluded from the analysis (quality of representation inferior to 0.2 for both populations).

^3^ *Perceived mastery* was excluded from the final model because its factor loading on the factor was inferior to 0.5.

### Determinants of risk perception

In both regions, being a woman increased risk perception. In Montérégie, having a university level of education and being 18–34 years of age decreased risk perception while having a higher level of knowledge of LD increased risk perception (Table 5). In Neuchâtel, living in a risk zone and knowing someone who had previously had LD, increased risk perception (Table 5).

**Table 5:** **Determinants of LD risk perception**

	Montérégie		Neuchâtel	
	n = 392		n = 406	
	Coefficient	95% CI	Coefficient	95% CI
Gender (Being a woman, man = reference category)	0.25***	(0.1-0.39)	0.26***	(0.11-0.40)
Age				
18-34 yr	-0.52***	(-0.74- -0.30)	-0.01	(-0.20-0.19)
35-54 yr	-0.16*	(-0.3--0.01)	0.04	(-0.13-0.21)
55+ yr (reference category)	0		0	
University diploma	-0.18*	(-0.34- -0.01)	-0.08	(-0.22-0.07)
High level of general knowledge on LD	0.37***	(0.18-0.57)	0.07	(-0.09-0.22)
Leaving in the higher risk area in Neuchâtel canton	-	-	0.18*	(0.03-0.33)
Knowing someone who had LD	0.14	(-0.25-0.53)	0.28***	(0.12-0.44)
r2	0.119		0.108	

*p < 0.05; ***p < 0.001.

## Discussion

Risk perception of LD has been studied in the past, mostly as a predictor of individual level preventive behavior along with knowledge and other factors. The vast majority of these studies were undertaken in the United States [19, 21, 23, 27–29, 32, 33, 45, 59], while more recent studies were done in the Netherlands [24, 25] and in the United Kingdom [22]. To our knowledge, this is the first study to measure risk perception regarding LD and its determinants in Canada and in Switzerland, and to address risk perception of LD in different epidemiological contexts with an international comparative perspective.

The populations of these two regions were different on several aspects. First of all, nearly half of the surveyed population in Montérégie had never heard about LD (46%), with only a minority of the participants (15%) demonstrating a good level of knowledge, whereas in Neuchâtel, more than 8 out of 10 people knew of the disease and close to 60% had a good level of knowledge of the disease. These differences may be due to several contextual factors including a longer experience with LD in Switzerland, where the disease is highly endemic, through public health messages, media coverage, social networks, personal history of infection and schools. Neuchâtel residents declared themselves as more often exposed through outdoor activities than in Montérégie, which also reflects the fact that most people in this region live near (if not ‘in’) the tick inhabited regions. Accordingly, the mean global risk perception score was higher in Neuchâtel. This is consistent with previous findings comparing risk perception in low and high incidence states in the United States and showing that risk perception was positively correlated to incidence of LD [28, 29].

Looking at the same results, we can also highlight that a lack of knowledge about the risks of LD still persists in Neuchâtel despite the high regional incidence: 22% of the respondents declared they had never heard about the disease, 35% did not know that the disease was transmitted by a tick and three out of four did not know the difference between LD and TBE. A previous national study in the United States also reported that 7% of people had never heard about LD in high-incidence States, and that 22% declared that they did not know how LD is contracted [29]. These results suggest that living in an endemic area established for a long time does not guarantee that the entire population will be aware of the risks and have sufficient knowledge of how to protect themselves. This underscores the need to adjust, strengthen and maintain communication efforts about LD risks even as the epidemiological situation evolves over time.

Some surprising findings arose. First, the mode of the *perceived regional susceptibility* was found to be equal between regions even though the incidence was nearly 200 times higher in Neuchâtel compared to Montérégie for this period (95 *vs* 0.5/100,000). In Montérégie, the population *perceived regional susceptibility* was greater than the expert’s *perceived regional susceptibility*, who most often consider the risk to be low in this region. One possible explanation for this observation could rest on the novelty of the hazard for the Montérégie population. New threats frequently lead to higher perceived risk in the general population, as has been previously demonstrated in studies comparing risk perception between different kinds of hazards [35].

Second, Montérégie respondents rated the risk for themselves (mode = 2, mean = 2.7) and the risk for the residents of their region (mode = 4, mean = 3.2) differently. The underestimation of the personal risk as opposed to the general population risk has been described before for other hazards and is known as ‘unrealistic optimism’ [39]. Explanations for this optimism have been extensively studied before and are reviewed in Shepperd and colleagues [60]. In Neuchâtel, this phenomenon is not observed. One possible explanation is that past history with LD among respondents or their relatives is more prevalent in this region. Personal experiences with a hazard has been shown to decrease unrealistic optimism [61].

This study showed that the perceived risk of LD differed between the population and their regional experts. In Montérégie, experts rated the measured components of risk as smaller and more ‘controllable’ than the population. Many studies have demonstrated differences between public and expert risk perception for other hazards [35, 38, 62] and this trend can be problematic when decisions have to be made about risk management options. Given that risk perception can affect the adoption of preventive behaviors, as well as the social acceptability of public health actions, our results suggest not only that risk perception of a hazard has to be taken into account when making such decisions, but also that risk perception should be measured directly in the target population, and cannot be extrapolated from studies carried out in different contexts, nor by regional experts. Because of the limited number of experts who participated in this study, statistical analysis could not be performed to compare perceptions between the population and experts and between both groups of experts.

One interesting aspect of this study lies in the use of EFA to build a global risk perception score for LD. Past studies of risk perception and LD have used individual perception variables such as the perceived susceptibility and the perceived severity of the disease as the dependant variable or as independent variables to predict preventive behaviors [25, 28, 29, 32]. However, we hypothesised that risk perception is a complex construct that can only be imperfectly captured by individual survey questions. Most individual perception variables are correlated, and factor analysis can be used to verify the internal consistency of a set of questions designed to measure a construct (internal consistency) and to reduce the measurement bias related to individual questions by identifying which composition of items best represents a single factor (composite reliability) [63]. Although identifying determinants of a global perception score could be more interesting for public health decision-making than focusing on individual perception variables, the use of EFA has been criticized, mainly because of the absence of objective criteria to guide decisions necessary to complete the analysis, particularly in the choice of the type of rotation of factors [64]. In this study, no rotations were performed in the final model given that only one factor was retained. We used EFA to explore which perception variables to include in a global risk perception score and we interpreted the results in light of previous findings. The Health Belief Model recognizes two main dimensions of risk perception: the severity of the hazard and the susceptibility of individuals to this hazard [31]. Empirical studies have underlined that individual susceptibility can be perceived differently than the susceptibility for the general population [39]. These three dimensions *(perceived severity, perceived individual susceptibility, perceived regional susceptibility*) were identified in this study, along with *feeling of worry*, as the main contributors to a factor with the EFA realized in both populations and this strengthens the choice of these four variables in the construction of a global score.

Another important result of our study was the identification of different determinants of risk perception regarding LD in both populations, suggesting that the determinants may not be universal but rather context-dependant. The only common predictor was gender, a well-known determinant of risk perception. Possible explanations for gender differences in risk perception have been explored in the risk perception literature and include differences in social roles and activities [65]. In Montérégie, the effect of age was also highlighted, where being less than 35 years old decreased risk perception. This effect has been demonstrated before for other hazards, particularly regarding risk perception of road accidents [66]. One interesting finding is that in Neuchâtel, where the disease has been endemic for a long time, the level of knowledge was not significantly associated with risk perception, in contrast to the Montérégie region. It is both the exposure (living in a high risk region) and past history with LD that constituted the strongest predictors of risk perception. Only a handful of other studies have previously identified determinants of LD risk perception, being that the main focus of these other studies has generally been to identify predictors of the adoption of preventive behavior. Knowledge of LD [24], knowing someone who has had LD [24], the presence of tick populations [59], and cultural identity [21] have been identified before as factors that may affect risk perception of LD.

Globally, these results suggest that in populations facing an emerging threat such as LD in Montérégie, risk perception is mostly determined by globally available information. In the Montérégie context of LD emergence, risk perception seemed less affected by an individual’s specific circumstance, i.e. their exposure and past history with LD, than it was in the LD endemic region of Neuchâtel. This further suggests that the availability of reliable information becomes particularly important in a context of emergence. This comparison can provide useful insights for both Canadian and Swiss decision-makers, as well as for other countries facing a challenge of LD emergence. On the one hand, this study provides important information for local populations and on the other hand, international comparisons may allow us to understand what might occur in future epidemiological contexts.

Nevertheless, this study presents some limitations. First, by recruiting participants through two web panels, the population samples were not probabilistic and were restricted to internet-users. Generalisation of the results should be interpreted in consequence. The mean response rate was considerably low. Previous Canadian studies using the same Canadian panel had response rates around 20 to 25% (Léger & Marketing, personal communication). A wide variety of factors are known to affect the response rate of web surveys, such as the methods of delivery [67]. For this study, the firm which administered the survey closed the survey access when 400 participants had completed the survey in a region, which took three days in Montérégie (response rate of 8.3%) compared to 12 days in Neuchâtel (response rate of 36%). A longer response period, especially in Montérégie, might have led to a better response rate. Though, these response rates depend in fact on the number of people who were initially contacted (5,222 in Montérégie vs 1,233 in Neuchâtel), and the mean response rate should be considered with regards to the recruitment process.

Secondly, all participants read a descriptive text before answering questions pertaining to perception. This was a strategic decision implemented in the study design with the objective of increasing the number of eligible respondents, particularly in Montérégie, where we expected that the majority of residents would not know enough about LD to complete the survey. But the content of this text may have altered participants’ perception and consequently, may have biased their ‘true’ perception (*i.e.* the perception they would have had without reading the text, influenced by the information they already had about the disease).

Another limitation is the cross-sectional design of this study. Measures of risk perception, such as psychometric variables, can change rapidly over time [63]. Future work should include additional administrations of the risk perception questionnaire in the same regions in order to provide insights on the temporal evolution of risk perception and their determinants in both populations, and to allow confirmation of the risk perception factor structures.

Finally, regression models revealed interesting determinants in both regions, but explained only 12% and 11% of the variance in Montérégie and Neuchâtel, respectively. Even if these percentages are low, they are consistent with other psychometric studies of risk perception [39]. When interpreting the multivariate analysis, we must keep in mind that several other possible unmeasured factors may have an impact on risk perception.

## Conclusion

This study underlined significant differences between the two populations of Montérégie and Neuchâtel and between the general public and their regional experts, and demonstrated interesting trends within these populations, which are important elements to consider when planning and implementing LD prevention activities. Results revealed the need to strengthen and maintain LD risk communication in both regions and may help to prioritize target groups for enhanced communication about LD risk, for example men of 18–34 years of age, who may be more frequently exposed through outdoors activities, tend to have a poorer level of knowledge of LD as well as a lower perception of risk. The findings of this study also reveal the importance of monitoring risk perception in the target population, as it is determined by various dynamic factors that vary according to specific contexts, and as risk perception of the general public tends to differ from that of experts. Moreover, re-assessing risk perception over time (for example after communication campaigns) or across regions likely to have heterogeneous beliefs about LD should be considered in order to better align public health preventive actions for LD with underlying determinants and to enhance the efficacy of these actions.
